# Cardiac and vascular changes in elderly atherosclerotic mice: the influence of gender

**DOI:** 10.1186/1476-511X-9-87

**Published:** 2010-08-19

**Authors:** Thiago MC Pereira, Breno V Nogueira, Leandro CF Lima, Marcella L Porto, Jose A Arruda, Elisardo C Vasquez, Silvana S Meyrelles

**Affiliations:** 1 Laboratory of Transgenes and Cardiovascular Control, Physiological Sciences Graduate Program, Health Sciences Center, Federal University of EspÃ­rito Santo, Av. Marechal Campos 1468, Vitoria, ES 29043-900, Brazil; 2 Research Center, Emescam College of Health Sciences, Vitoria, ES, Brazil

## Abstract

**Background:**

Although advanced age is considered a risk factor for several diseases, the impact of gender on age-associated cardiovascular diseases, such as atherosclerotic processes and valvular diseases, remains not completely clarified. The present study was designed to assess aortic valve morphology and function and vascular damage in elderly using the apolipoprotein E knockout (ApoE KO) mouse. Our hypothesis was that advanced age-related cardiovascular changes are aggravated in atherosclerotic male mice.

**Methods:**

The grade (0 to 4) of aortic regurgitation was evaluated through angiography. In addition, vascular lipid deposition and senescence were evaluated through histochemical analyses in aged male and female ApoE KO mice, and the results were compared to wild-type C57BL/6J (C57) mice.

**Results:**

Aortic regurgitation was observed in 92% of the male ApoE KO mice and 100% of the male C57 mice. Comparatively, in age-matched female ApoE KO and C57 mice, aortic regurgitation was observed in a proportion of 58% and 53%, respectively. Histological analysis of the aorta showed an outward (positive) remodeling in ApoE KO mice (female: 1.86 ± 0.15; male: 1.89 ± 0.68) using C57 groups as reference values. Histochemical evaluation of the aorta showed lipid deposition and vascular senescence only in the ApoE KO group, which were more pronounced in male mice.

**Conclusion:**

The data show that male gender contributes to the progression of aortic regurgitation and that hypercholesterolemia and male gender additively contribute to the occurrence of lipid deposition and vascular senescence in elderly mice.

## Background

Although several studies have characterized advanced age as a risk factor for cardiovascular diseases [1,2], the impact of gender on age-associated cardiovascular performance remains to be completely delineated [3,4]. This question is important because gender-related differences in cardiovascular aging, such as atherosclerotic processes and valvular diseases, may help to explain, in part, the greater longevity of females [5,6].

Over the past few decades, the availability of new investigative tools, including the homozygous apolipoprotein E knockout (ApoE KO) mouse, has contributed to understanding the atherosclerotic process and cardiovascular diseases [7,8]. ApoE is a constituent of VLDL synthesized by the liver, mediates high-affinity binding of ApoE containing lipoprotein particles to LDL receptors, and is responsible for the cellular uptake of these particles [9]. Therefore, the ApoE KO mouse develops marked hypercholesterolemia and spontaneous atherosclerosis [9-11]. In this experimental model, the influence of gender has been reported only with Western-type cholesterol-rich diets in young adult mice [12,13], but the impact of both elderly and gender on cardiovascular function has not been elucidated.

In the present study, our hypothesis was that advanced age affects the cardiovascular structure of atherosclerotic mice, which could be aggravated in males. On a normal chow diet, ApoE KO mice were subjected to *in vivo *angiography and to *in vitro *histochemical analysis to characterize valve and aortic morphology.

## Materials and methods

### Animals

Aged male and female mice (18 months old) were randomly divided into four groups: C57 (females: n = 26; males: n = 22) and ApoE KO (females: n = 28; males: n = 23). Animals were obtained from the animal facilities of the Laboratory of Transgenes and Cardiovascular Control at the Federal University of Espirito Santo. Mice were fed a normal chow diet and water *ad libitum *and were housed separately in temperature-controlled rooms (22°C) under a 12 h light/dark cycle. All procedures were conducted in accordance with the institutional guidelines for animal research and the protocols were previously approved by the Institutional Ethics Committee for Use of Animals (CEUA 001/2009).

### Angiographic Analysis

Mice were anesthetized with ketamine/xylazine (91.0/9.1 mg/kg, IP) and polyethylene catheters (PE10 - PE50) filled with heparinized (10 UI/mL) saline were inserted into the right carotid artery for the contrast agent infusion. Each mouse was placed in a supine position to obtain images at 0° and 45° with an angiographic X-ray System (Shimadzu Corporation, Japan) at a speed of 5 frames/s. The X-ray angiography was performed with a manual injector that was controlled to reproducibly deliver 0.15 mL/s of non-ionic contrast media containing 35% iodine (Telebrix; Guerbet, France) for 3 s. Serial images of each mouse were then recorded and the aorta internal diameter analyzed by quantitative coronary analysis software (QCA, Shimadzu Corporation, Japan). Detection of aortic regurgitation (AR) was evaluated by intensity, extension and persistence of contrast after successive systoles and classified by grades of severity (0 to 4) based on the study of Pujadas [14].

### Cardiopulmonary parameters

The wet weight of the lung was measured and the tissue was then placed in an oven at 60°C for 24 h. The lungs water content (%H_2_O) was determined as follows [15]: %H_2_O = (wet weight - dry weight)/wet weight × 100. Cardiac wet weight was used to determine the cardiac weight/body weight ratio.

### Histological Valve and Vessel Processing

At the end of the experiments, mice were euthanized with sodium thiopental overdose (100 mg/kg, IP) and perfused via the left ventricle with phosphate-buffered saline (PBS, pH 7.4; 0.1 M) followed by a fixative solution of formaldehyde (4%) at a pressure of 100 mmHg. After remaining overnight in the fixative solution, the perivascular adipose tissue of the aorta was removed. The aortic valve and the portion of the ascendant aorta were embedded in a 24% gelatin solution and then cross-sectioned at a 10-μm thickness using a cryostat (Jung CM1800; Leica, Wetzlar, Germany). For each animal, aorta cross-sections were mounted on gelatin-coated slides and stained with Oil-Red-O (Sigma-Aldrich, St. Louis, MO, USA) for neutral lipids detection and stained with hematoxylin (Sigma-Aldrich) for valve morphological analysis which was done by an independent investigator. Extreme care was taken in sectioning the heart so that the valves were cut transversely. For longitudinal analysis, the hearts were embedded in paraffin and cut on a longitudinal plane with sections of 8-12 μm thickness. The slides were stained with hematoxylin-eosin for detection of cellularity and thickness. The slides were also stained with Masson's trichrome for detection of the presence of fibrosis and with von Kossa for calcification analysis. The remnant whole aorta was opened lengthwise and stained *en face *for lipid deposition and vascular senescence activity.

### Senescence Activity β-Gal Staining

Staining for senescence by β-gal activity was performed as described by Minamino et al. [16]. Briefly, *en face *aortic samples were incubated for 24 h at 37°C in freshly prepared β-gal staining solutions (pH 6.0) containing 2.4 mM 5-bromo-4-chrolo-3-indlyl-D-galactopyranoside (X-gal, Sigma Aldrich), 4.7 mmol/L potassium ferrocyanide, 4.9 mmol/L potassium ferricyanide, 150 mmol/L NaCl, 1 mmol/L MgCl_2 _and 40 mmol/L citric acid. The senescence analysis was performed by detection of blue color produced by the enzymatic reaction and the images were captured with a photographic camera (Canon, USA). The quantification of senescence was performed using an National Institutes of Health (NIH) Image program (Image-J 1.35 d, NIH, Bethesda, USA) and the examiner was blinded to the experimental groups.

### Morphometry

Images of the aorta and the valve were captured with a color video camera (VKC150, Hitachi, Tokyo, Japan) connected to a microscope (Olympus AX70, Olympus, Center Valley, PA, USA). Analysis was conducted with a Leica image program (2100 Leica EWS; Leica, Wetzlar, Germany) and an NIH Image program by an examiner blinded to the experimental groups. The program was calibrated with a graduated slide. By using a 4× objective, the vessel cross-sectional area (V_CSA_) and the lumen cross-sectional area were calculated. The vascular remodeling ratio was obtained by dividing each animal's V_CSA _by the average V_CSA _of the C57 group and each sample was classified as absence (0.95-1.05), inward (<0.95), or outward (>1.05) remodeling. In the left ventricle, the maximum thickness of the valvule and the width of at least 10 cardiomyocytes were measured for seven mice from each group.

### Measurement of Plasma Cholesterol Levels

A blood sample (200 μL) was taken from the carotid artery of each animal and the plasma total cholesterol was measured using a commercial colorimetric kit (Bioclin, Belo Horizonte, Brazil).

### Statistical Analysis

Continuous variables are presented as mean ± SEM. Statistical analysis was performed with a one-way analysis of variance (ANOVA), followed by the Tukey *post hoc *test for multiple comparisons of continuous variables between groups. Student's *t*-test for independent samples was used when appropriate. Categorical variables are presented as frequencies and were compared using the Fisher's exact test. The significance level was set at p < 0.05.

## Results

Table 1 summarizes the average values of body and cardiac weight, cardiac myocyte size, pulmonary water content and plasma cholesterol levels. At the time of the experiments, body weight did not differ between C57 and ApoE KO groups. As expected, total plasma cholesterol levels were significantly higher in ApoE KO females (4.1-fold, p < 0.001) and males (6.7-fold, p < 0.001) than in females and males of the C57 group. Plasma cholesterol levels were significantly augmented (93%; p < 0.001) in aged male compared with aged female ApoE KO mice. Based on cardiac weight/body weight ratio and myocyte size, cardiac hypertrophy was not observed in male as compared to female animals in either C57 or ApoE KO groups. Also, an increase in pulmonary water content was not observed between groups.

**Table 1:** Body and cardiac weight, cardiomyocyte size, pulmonary water content and plasma cholesterol levels of C57 and ApoE KO.

	Groups			
	
Parameters	C57		ApoE KO	
	
	Female	Male	Female	Male
Body weight (g)	31 ± 0.9	33 ± 0.7	30 ± 0.5	32 ± 0.7
Cardiac weight/body weight ratio (mg/g)	5.9 ± 0.4	5.7 ± 0.4	6.0 ± 0.4	5.9 ± 0.3
Cardiomyocytes width (μm)	15 ± 0.1	15 ± 0.2	15 ± 0.1	15 ± 0.1
Pulmonary water content (%)	76 ± 0.6	76 ± 1.2	75 ± 0.5	76 ± 0.9
Plasma cholesterol (mg/dL)	81 ± 4	96 ± 7	336 ± 32*	650 ± 92^# ^*

Values are means ± SEM. 7 to 11 animals per group.

*p < 0.001 vs C57 group and ^#^p < 0.001 vs female (ANOVA)

**Figure 1:**
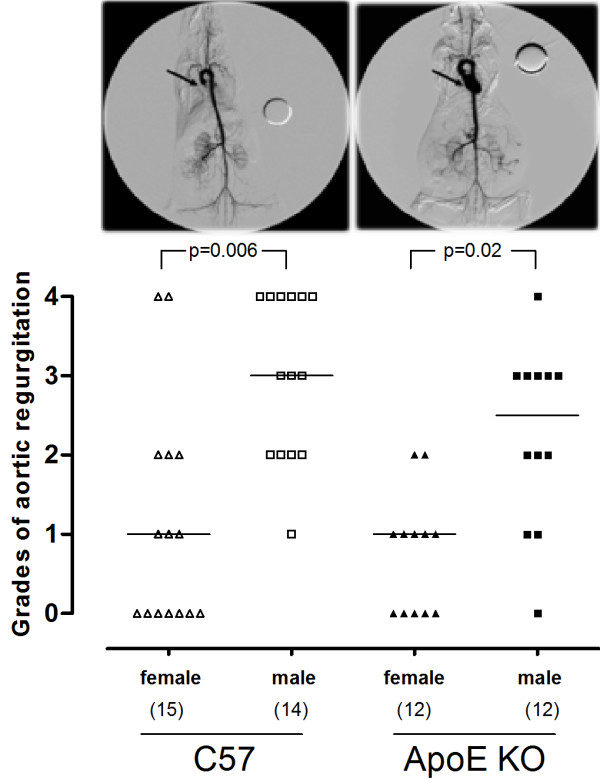
**Influence of gender on aortic regurgitation in aged normocholesterolemic and hypercholesterolemic mice. **Top panel: typical angiographies showing aortic regurgitation in the male (right image) but not in the female (left image). Bottom panel: scatter plot showing the severity of aortic regurgitation (grades: 0 = none, 1 = light, 2 = mild, 3 = moderate, 4 = severe) in males compared with females in both C57 and ApoE KO groups. Horizontal bars represent the median for each group. p values were calculated using the Fisher's exact test and indicate statistical significance between proportions. The number of animals per group is shown in parentheses.

Using angiography with injection of iodized contrast, we found a remarkable aortic regurgitation in aged ApoE KO and C57 males compared with aged females (Figure 1, bottom panel). The proportion of male mice (96%) with aortic regurgitation (grades 1-4) was significantly higher than of female mice (51%) when grouping ApoE KO and C57 animals (p = 0.0008). When the analysis was restricted to the normocholesterolemic C57 group the proportion of animals with aortic regurgitation was significantly higher in males than in females (100% vs 53%; p = 0.006). Similar results were observed in the hypercholesterolemic ApoE KO group (92% vs 58%; p = 0.02). A typical angiography of an aged female without aortic regurgitation (left image) and a typical angiography of an aged male with (right image) aortic regurgitation are shown in the top panel of Figure 1.

**Figure 2:**
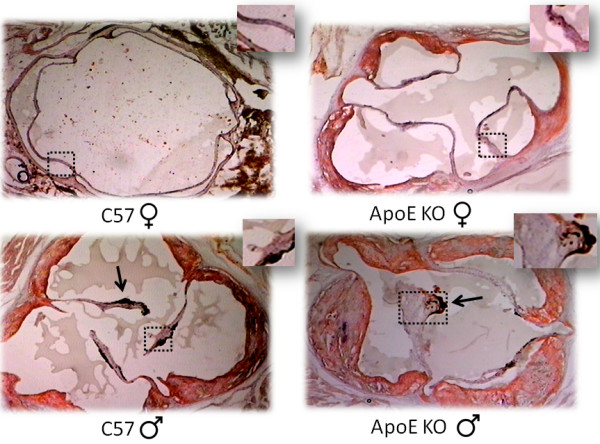
**Typical photomicrographs of the aortic valves of male and female mice from the normocholesterolemic C57 and hypercholesterolemic ApoE KO groups. **Squares indicate the maximum thickness of each valve. Arrows indicate valvar lesions in males of both C57 and ApoE KO groups as indicated by hemosiderin deposition. The sections were stained with both Oil-Red-O and hematoxylin. Magnification: 400×.

**Figure 3:**
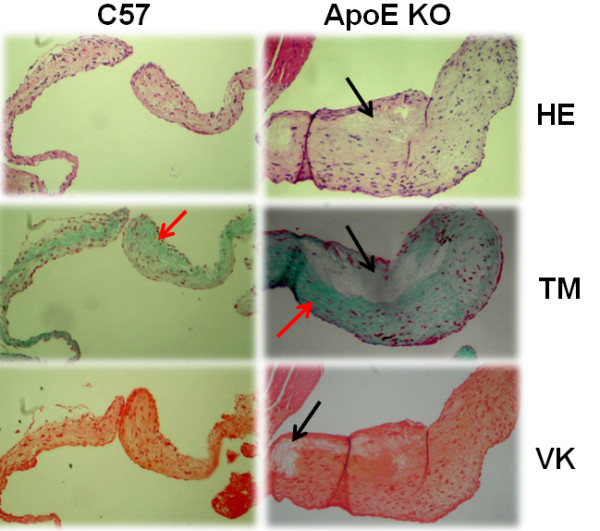
**Typical photomicrographs of aortic valves on a longitudinal axis from a male aged hypercholesterolemic ApoE KO and a normocholesterolemic C57 mouse. **Samples were stained with hematoxylin-eosin (HE), Masson's trichrome (MT), and von Kossa (VK). Black arrows indicate diffuse acellularity and myxoid thickening of the spongy layer (HE, MT and VK) in the ApoE KO mouse. Red arrows indicated valvular fibrosis with dense collagen (MT) mainly in the ApoE KO mouse. Magnification: 250×.

Figure 2 illustrates the results of histological examinations of the heart showing the thickening of aortic valves in aged males compared with females of the C57 group (106 ± 4 vs 32 ± 5 μm, respectively; p < 0.001) and, more significantly, in aged males compared with females of the ApoE KO group (186 ± 3 vs 69 ± 6 μm, respectively; p < 0.001). The analysis of longitudinal valvular sections of aged males (Figure 3) showed acellularity, glycosaminogycan transformation and fibrosis of the sponge layer of the leaflet mainly in ApoE KO mice. Valvular calcifications were not observed in C57 or ApoE KO animals.

**Figure 4:**
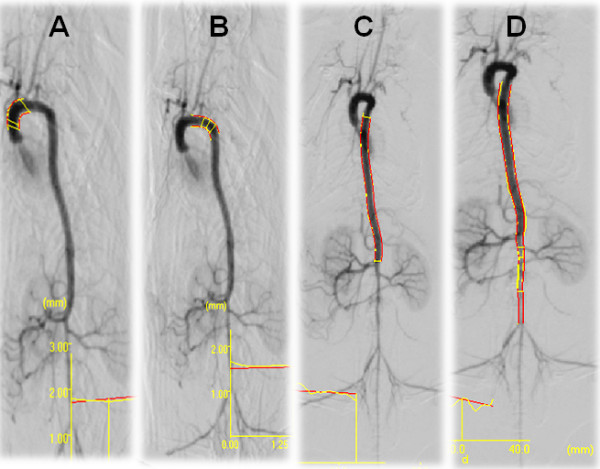
**Typical angiography analysis of the internal diameter (parallel red lines) of the aorta divided into four segments: **A) ascendant aorta - left common carotid; B) left common carotid - descendant thoracic portion of aorta; C) descendant thoracic portion of aorta - right renal artery; D) descendant thoracic portion of aorta - bifurcation of the common iliac artery. For a better visualization, A and B images were performed at an angle of 45° whereas C and D were at 0°.

The angiographic analysis in the four segments of the aorta did not show significant differences in the internal diameter between groups as illustrated in Figure 4.

**Figure 5:**
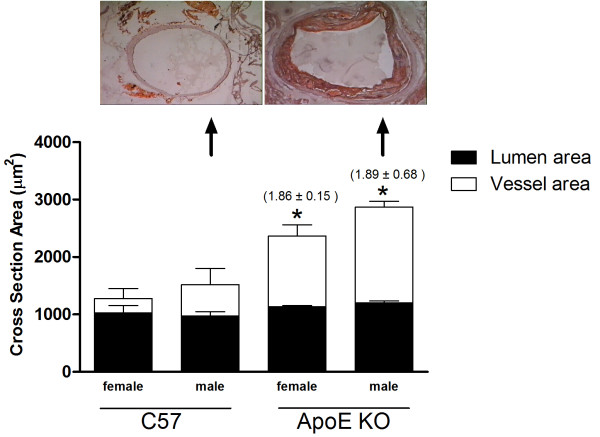
**Top panel: typical photomicrographs of cross sections of ascending aorta showing an augmented vessel diameter in the hypercholesterolemic ApoE KO compared with the normocholesterolemic C57 mouse. **The sections were stained with both oil-red-O and hematoxylin (magnification: 400×). Bottom panel: Bar graphs showing average vessel (white bars) and lumen (black bars) cross-sectional areas of C57 (n = 6) and ApoE KO (n = 6) mice. Values are mean ± SEM. Numbers in parentheses indicate a positive remodeling ratio (>1.05). *p < 0.05 compared with vessel area of C57 groups.

Figure 5 (bottom panel) summarizes the data of the cross section area of the ascending aorta, showing a similar lumen area in all groups. However, the vessel area was significantly increased in female (2364 ± 192 μm^2^) and male (2869 ± 102 μm^2^) ApoE KO mice, compared with C57 mice (1274 ± 175 and 1517 ± 283 μm^2^, respectively; p < 0.05). Consequently, a positive (>1.05) vascular remodeling was observed in both female (1.86 ± 0.15) and male (1.89 ± 0.68) ApoE KO mice, using the C57 animals as reference values. The top panel of Figure 5 shows typical photomicrographs of ascending aorta cross sections, indicating an outward vascular remodeling in an ApoE KO compared with a C57 mouse.

**Figure 6:**
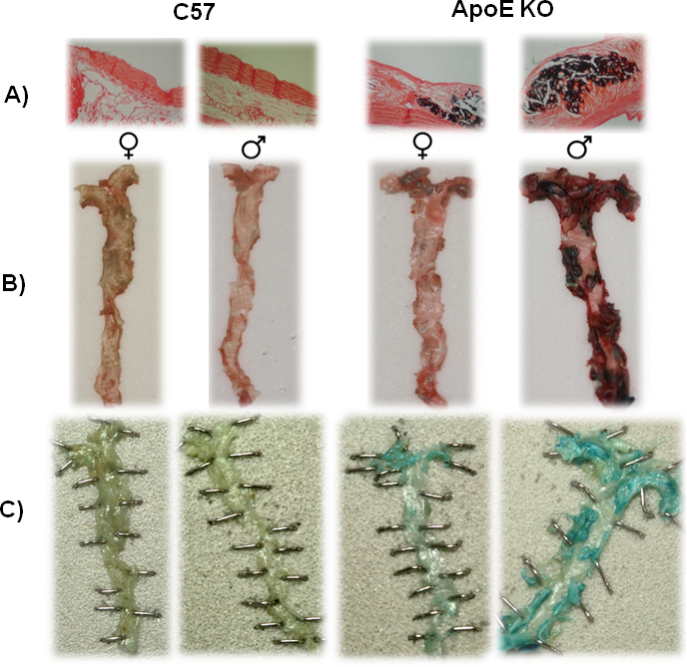
**Morphological analysis of aortas in both genders of normocholesterolemic C57 and hypercholesterolemic ApoE KO mice. **(A) typical micrographs showing the severity of calcium deposition (Von Kossa staining; magnification: 250×) and e*n face *images of (B) lipid deposition (Oil-Red-O staining) and (C) vascular senescence (X-gal, pH 6.0), in ApoE KO mice mainly in the male but not in either male or female C57 mice.

In the histochemical evaluation of the aorta (Figure 6), calcium deposition was observed only in the ApoE KO group and mainly in male animals (panel A). *En face *whole aorta analysis (panel B) showed greater lipid deposition in female and male ApoE KO mice (0.21 ± 0.04 and 0.35 ± 0.05 cm^2^) when compared with C57 mice (0.11 ± 0.01 and 0.12 ± 0.01 cm^2^, respectively) (p < 0.05). Panel C shows representative *en face *analysis of vascular senescence (β-gal positive cells). An increase in stained area was observed in the aorta of female and male ApoE KO mice (0.025 ± 0.02 and 0.19 ± 0.08 cm^2^, respectively) as compared with C57 mice (0.010 ± 0.008 and 0.016 ± 0.009 cm^2^, respectively) (p < 0.05).

## Discussion

This study describes the functional and structural valvular and vascular changes by gender in normal and hypercholesterolemic aged mice. We observed that male gender is an important factor for the progression of aortic regurgitation in both normocholesterolemic (C57) and hypercholesterolemic (ApoE KO) mice. In addition to male gender, hypercholesterolemia also contributes to the occurrence of vascular senescence in elderly mice. In contrast, the outward (positive) remodeling observed in aortas of ApoE KO mice was not influenced by gender.

Diagnostic imaging methods used for investigating human cardiovascular diseases have also been used in rats [17] and with limitations in mice [8,18]. While measuring the internal diameter of the aorta through angiography, we incidentally observed aortic regurgitation in elderly male mice. Age-related valvular changes and echocardiographic parameters have only been described in a few studies [19-21] but the influence of gender on those parameters was not investigated. To our knowledge, this study is the first to report a pronounced aortic regurgitation in aged male mice compared to age-matched females, independent of cholesterol levels. This finding does not appear to be due to estradiol protection as this result was also confirmed in ovariectomized females (data not shown). Moreover, we must consider that the observed sex differences may not simply be reflective of differences in levels of circulating hormones [22].

Aortic regurgitation results from malcoaptation of the aortic leaflets due to abnormalities [8]. In our study, the observed aortic regurgitation was associated with an increase in valvular thickness. This has also been observed in other experimental rodent models [17,19,23] and humans [24]. Although aortic regurgitation is a chronic disease characterized by cardiac volume overload that induces progressive cardiac hypertrophy and eventually contractile dysfunction [25,26], neither compensatory cardiac hypertrophy nor pulmonary water content were observed. This finding is in agreement with studies done in humans showing that aortic regurgitation often remains asymptomatic for decades before the development of heart failure [17,27]. It has also been shown that valvular regurgitation could be influenced by the type of anesthetic used, due to an effect on left ventricular ejection [28,29]. In this study we used a ketamine and xylazine mixture, the most commonly used anesthetic during image analysis in mice [28,30]. Although it has been described that this anesthetic could influence aortic regurgitation by reducing heart rate [19,23], in our study, males and females had similar chronotropic activity after the catheterization procedure.

Valvulopathy usually occurs mainly in left-sided valves, particularly the aortic valve, which could be sequentially due to mechanical stress, cusp thickening, inflammation and decrease of cusp pliability, leading to regurgitation [31]. In agreement with the above study, we also observed cusp thickening, collagen deposition, acellularity and aortic regurgitation in aged male mice of both groups. In addition, the influence of both gender and hypercholesterolemia on valvular lesions has been reported only in epidemiological studies [6,32]. Our data show, for the first time, that male gender and hypercholesterolemia additively contribute to valvular degeneration without aggravation of aortic regurgitation.

In our study, the angiography also revealed the maintenance of aorta internal diameter in both C57 and ApoE KO groups. *Ex vivo *analysis confirmed this finding and showed an aorta outward remodeling. The vascular remodeling of large arteries is considered an adaptive structural change observed in both clinical [33-35] and experimental [7,34,36] studies in response to a variety conditions, such as atherosclerosis, hypertension and lumen obstruction. Aortic remodeling in ApoE KO mice is not established in young animals [7,37] but is detected by 13 months of age [38]. In our study, we observed that 18-month-old ApoE KO mice still preserve the aortic vessel lumen compared with normal animals (C57). Because overcompensation is a feature of the remodeling response in human coronary arteries [39], we suggest, as have others [33,40], that the elderly ApoE KO mouse constitutes a good model for studies on advanced stages of remodeling mechanism of large arteries.

Even though aortic remodeling was found to be similar for both genders in ApoE KO mice, vascular senescence and lipid deposition were influenced by male gender. Senescent cells show progressive telomere shortening [41], negative gene expression regulators [42], decreased expression of vasodilators [43,44] and increased proinflammatory molecules [16], contributing to both progression of atherosclerosis and pathogenesis of vascular aging [45,46]. In this study, we evaluated senescence through β-gal activity, which is considered to be one of the best markers currently available. In agreement with other studies [41,42], we observed that hypercholesterolemia promotes inflammation and vascular senescence. The novelty in this study is the finding that male gender and hypercholesterolemia have additive effects on vascular senescence and vessel lipid deposition. Although estrogens seem to slow the senescence through activation of estrogen receptors [22,47], ovariectomy did not cause vascular senescence in C57 female mice and did not aggravate it in ApoE KO females (data not shown). In addition, ovariectomy did not modify plasma cholesterol levels. Based on other findings [48], we hypothesized that endogenous and/or genetic factors could be essential for the development of vascular senescence.

*En face *aorta images demonstrated that the localization of lipid deposition and vascular senescence in aged ApoE KO mice was mainly in the aortic arch, which is a turbulent flow area. This finding corroborates the hypothesis from others [42,46,49] that sites of high endothelial cell turnover associated with atherogenic stimuli can promote telomere shortening and possibly induction of senescence. We also could expect that oxidative stress and DNA damage contribute to vascular senescence (or vice-versa) and thus further promote atherogenesis.

In conclusion, both normocholesterolemic (C57) and hypercholesterolemic (ApoE KO) elderly mice exhibit aortic regurgitation, but it is of higher proportion in males than in females. The notable proportion of females with absence of aortic regurgitation leaves open the possibility of a gender-specific protection mechanism. On the other hand, vascular outward (positive) remodeling observed in hypercholesterolemic mice does not differ between the genders. Interesting that only vascular lipid deposition and senescence in elderly mice are influenced by both hypercholesterolemia and male gender, highlighting sex differences in the progression of vascular diseases in this important model of spontaneous atherosclerosis. Although further studies are needed to reveal the mechanisms underlying these findings, the present data provide significant progress in the understanding of gender-specific aspects influencing the development of cardiovascular diseases in order to improve prevention, diagnosis and develop appropriate interventions in elderly women and men.

## Competing interests

The authors declare that they have no competing interests.

## Authors' contributions

TMCP carried out the animal experiments, analysis of the data, statistics and drafted the manuscript. BVN contributed to blood sampling and tissues sample pathology. LCF and MLP contributed to histology and senescence assay. JAA contributed to the study design and supervised the angiography analysis. ECV conceived the study design and prepared the manuscript. SSM conceived the study, participated in its design and supervision and in the critical revision of the manuscript. All authors read and approved the final manuscript.
